# Improved Bioavailability and High Photostability of Methotrexate by Spray-Dried Surface-Attached Solid Dispersion with an Aqueous Medium

**DOI:** 10.3390/pharmaceutics13010111

**Published:** 2021-01-16

**Authors:** Bhupendra Raj Giri, Jung Suk Kim, Jong Hyuck Park, Sung Giu Jin, Kyeong Soo Kim, Fakhar ud Din, Han Gon Choi, Dong Wuk Kim

**Affiliations:** 1 College of Pharmacy, Research Institute of Pharmaceutical Sciences, Vessel-Organ Interaction Research Center (VOICE, MRC), BK21 FOUR Community-Based Intelligent Novel Drug Discovery Education Unit, Kyungpook National University, Daegu 41566, Korea; giribhupen77@gmail.com; 2 College of Pharmacy, Hanyang University, Ansan 15588, Korea; jay910612@daum.net (J.S.K.); parkingjong@naver.com (J.H.P.); 3 Department of Pharmaceutical Engineering, Dankook University, Cheonan 31116, Korea; sklover777@dankook.ac.kr; 4 Department of Pharmaceutical Engineering, Gyeongnam National University of Science and Technology, Jinju 52725, Korea; soyoyu79@gntech.ac.kr; 5 Department of Pharmacy, Quaid-I-Azam University, Islamabad 45320, Pakistan; fudin@qau.edu.pk

**Keywords:** methotrexate, solid dispersion, solubility, bioavailability, photostability

## Abstract

Low aqueous solubility and poor bioavailability are major concerns in the development of oral solid-dosage drug forms. In this study, we fabricated surface-attached solid dispersion (SASD) to enhance the solubility, bioavailability, and photostability of methotrexate (MTX), a highly lipophilic and photo-unstable drug. Several MTX-loaded SASD formulations were developed for spray-drying using water as the solvent, and were investigated for their aqueous solubility and dissolution kinetics. An optimized ternary SASD formulation composed of MTX/ sodium carboxymethyl cellulose (Na-CMC)/sodium lauryl sulfate (SLS) at 3/0.5/0.5 (*w*/*w*) had 31.78-fold and 1.88-fold higher solubility and dissolution, respectively, than MTX powder. For SASD, the in vivo pharmacokinetic parameters AUC and C_max_ were 2.90- and 3.41-fold higher, respectively, than for the MTX powder. Solid-state characterizations by differential scanning calorimetry and X-ray diffraction revealed that MTX exists in its crystalline state within the spray-dried SASD. The MTX-loaded SASD formulation showed few physical changes with photostability testing. Overall, the results indicate that the spray-dried MTX-loaded SASD formulation without organic solvents enhances the solubility and oral bioavailability of MTX without a significant deterioration of its photochemical stability.

## 1. Introduction

**Figure 1:**
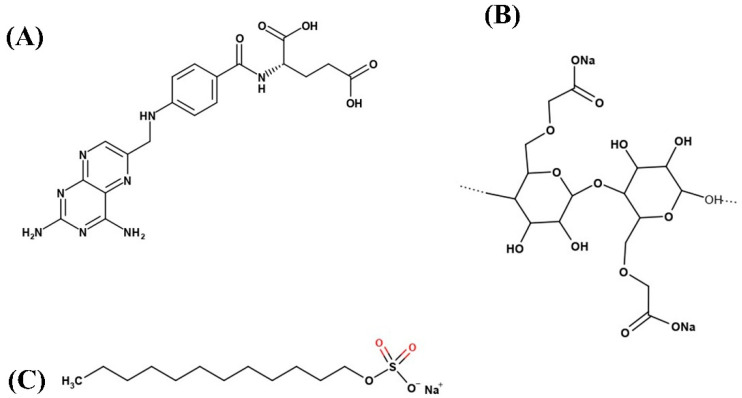
Chemical structure of: (**A**) methotrexate (MTX); (**B**) sodium carboxymethyl cellulose (Na-CMC); and (**C**) sodium lauryl sulfate (SLS).

Methotrexate (MTX, Figure 1A), chemically known as 2,4-diamino-N10-methyl propyl glutamic acid (MW: 454.5 g/mol, C_20_H_22_N_8_O_5_), is a commonly-used antifolate and antineoplastic drug. MTX is known to inhibit the metabolism of folic acid and interfere with the synthesis of DNA, RNA, and proteins [1,2]. It is clinically indicated for the treatment of autoimmune diseases such as rheumatoid arthritis and psoriasis, inflammatory diseases such as Crohn’s, and various cancers, including head and neck, breast, lung, lymphoma, and colorectal cancer [3,4]. Although MTX is in the limelight as a potent chemotherapeutic agent, its poor aqueous solubility (0.01 mg/mL at 20 °C) and low permeability result in suboptimal bioavailability (~18% for dose >40 mg/m^2^), which limits its clinical significance [5,6,7]. Due to its hydrophobic nature and low permeability, MTX is categorized as a class IV molecule in the Biopharmaceutical Classification System (BCS). Furthermore, MTX is chemically unstable; it rapidly decomposes when subjected to any light, or extremes of pH or temperature [8,9,10]. For these reasons, the development of appropriate oral dosage formulations for highly lipophilic and photo-unstable drugs like MTX remains challenging.

Drugs designed for oral administration must have adequate solubility in gastrointestinal (GI) fluids in order to reach their desired concentration in the systemic circulation if they are to exert any pharmacological activity. For BCS class II and class IV compounds, poor drug solubility results in rate-limited dissolution and slow absorption from the GI tract, leading to suboptimal oral bioavailability and inferior therapeutic efficacy with large inter-patient fluctuations in plasma concentrations [11]. Several approaches were investigated in order to resolve these challenges over the past few decades, particularly with BCS class II and IV drugs including cyclodextrin complexations [12], solid dispersion [13,14], lipid-based formulations [15], salt formulations [16,17], pro-drugs [18,19], and particle size reduction [20], etc. Solid dispersion (SD) is a widely employed and well-established method of enhancing solubility and dissolution. With SD, the hydrophilic carriers dissolve the hydrophobic drug and release it to the dissolving medium as fine particles (micro-nano-size range) in order to increase their surface area, which increases dissolution and therefore oral bioavailability [13,21]. Common SD methods require large volumes of an organic solvent or mixtures of organic solvents at the initial phase in order to dissolve the drug and carrier/s, followed by the removal of the solvents through spray-drying [22,23], freeze-drying [24], spray-freeze-drying [25], spray congealing [26], rotary evaporation [27], or hot-plate-drying [28]. However, the use of organic solvent/s is problematic, with safety and regulator concerns having been raised due to the solvents’ high toxicity, environmental hazards, and residue. Alternative SD methods such as fusion and melting require high heat, and thus cannot be used with thermolabile drugs [29,30].

The use of water, rather than organic solvents, for the SD of drugs with poor water solubility is safer, and has low environmental toxicity and reduced manufacturing costs. The in vitro/in vivo performance of hydrophobic compounds is shown to be improved when the crystalline drug is dispersed in an aqueous medium containing hydrophilic carriers in order to develop crystalline or amorphous SD [31,32,33,34]. In general, the crystalline compounds are physically and/or chemically more stable than their counterpart, amorphous forms. Given the poor photostability of MTX, the surface-attached solid dispersion (SASD) of MTX without a crystalline change could be an effective formulation option, potentially leading to improved solubility, enhanced bioavailability, and high photostability. Several prior efforts have focused on the fabrication of highly bioavailable dosage forms for MTX through different particle engineering techniques [7,35,36]; however, spray-dried MTX-loaded SASD formulations fabricated using an aqueous medium are rarely explored.

Therefore, the crystalline SASD of MTX was developed with a spray-drying technique in an aqueous medium as a way to improve the solubility, bioavailability, and photostability of the poorly water-soluble and photo-unstable drug MTX. Several spray-dried SASD formulations were fabricated with the drug, with a relatively low amount of sodium carboxymethyl cellulose (Na-CMC) as a hydrophilic polymer and sodium lauryl sulfate (SLS) as a surfactant, and were investigated using aqueous solubility, dissolution, and photostability tests. The morphological and physicochemical properties of the drug, carriers, and optimized formulation were assessed using different analytical tools. Lastly, the pharmacokinetic evaluation of MTX following the oral administration of MTX or MTX-loaded SASD was carried out in rats.

## 2. Material and Methods

### 2.1. Materials

MTX of >99% purity was obtained from Huzhou Zhanwang Pharm. Co. (Huzhou, China). Na-CMC (Aqualon^TM^ CMC), SLS (Kolliphor^®^ SLS) and all of the other pharmaceutical excipients with a purity of 99% or more were generously provided by Hanmi Pharm. Co. (Suwon, South Korea). The chemicals and solvents were of reagent grade, and were used as received, without additional purification.

### 2.2. Solubility Study

In order to select the appropriate carriers for the SASD, various hydrophilic polymers and surfactants were evaluated for their aqueous solubility with the drug. The drug was added in excess to 1 mL aqueous solution of each polymer and surfactant. The suspension was vortexed for a few seconds, and the microtubes were placed in an isothermal water bath shaker maintained at 25 °C and 100 rpm for 5 days in order to achieve the maximum solubilization. The samples were then centrifuged at 10,000× *g* for 10 min in order to remove the undissolved drug, and the supernatant was removed and diluted sufficiently with a mobile phase solution. The MTX concentration in the samples (10 μL) was quantified by HPLC (Agilent 1260 Infinity, Agilent Technologies, Santa Clara, CA, USA) equipped with Chem Station software (version B.04.02), a G1311C 1260 Quat pump, and an Inertsil ODS-4 column (GL Sciences, Japan, 250 mm × 4.6 mm I.D., 5 μm) maintained at 25 °C. The mobile phase comprised a 74/26 volume ratio of 0.1 M dibasic phosphate (pH adjusted to 3.0 with HCl solution) and methanol, respectively, eluted at a flow rate of 1 mL/min and monitored by a G1314B 1260 VWD VL detector at 303 nm.

### 2.3. Preparation and Optimization of the Surface-Attached Solid Dispersion

The polymer and surfactant with the highest aqueous drug solubility were dissolved in water (300 mL); a fixed quantity of MTX was added with continuous magnetic stirring for several minutes. The resulting suspension was spray-dried using a laboratory-scale Büchi B-290 nozzle-ype mini spray dryer (Büchi Co.; Flawil, Switzerland) with inlet and outlet temperatures of 130 °C and 75–85 °C, respectively; a flow rate of 5 mL/min; an aspiration of 100% (−45 mbar); and air pressure of 4 kg/cm^2^. A total of eight SDs were prepared (Table 1). These spray-dried SDs were tested for their aqueous solubility, as described above, and for in vitro drug dissolution. For the physical mixture (PM), MTX/Na-CNC/SLS at 3/0.5/0.5 (*w*/*w*) was mixed in a mortar and pestle in order to obtain a homogeneous mixture.

### 2.4. In Vitro Drug Release Study

The prepared formulations were investigated for their in vitro drug dissolution using a USP-type II dissolution apparatus (Vision Classic 6, Hanson Research Co., Los Angeles, CA, USA). The free drug, PM, and formulation powder, each equivalent to 50 mg MTX powder, were put into hard gelatin capsules (size 0), which were then subjected to dissolution testing. The dissolution apparatus baskets were filled with 900 mL distilled water maintained at a constant temperature of 37 °C ± 0.5 °C in an outer water bath with 100 rpm paddle rotations. In total, 1 mL dissolution medium was withdrawn at predetermined time intervals (0, 5, 10, 20, 30, 45, and 60 min), with an equivalent volume of fresh medium being immediately added into the baskets to compensate for the sampling loss. The samples were then filtered through a nylon syringe filter (0.45 μm), and the concentration of MTX in the filtrate (10 µL) was quantified by HPLC, as described above.

### 2.5. Characterization of the Solid SASD

#### 2.5.1. Scanning Electron Microscopy (SEM)

The physical morphology of the MTX powder, carriers, and MTX-loaded SASD formulation were assessed using a Hitachi S-4800 SEM (Tokyo, Japan). These samples were affixed onto a brass disk with double-sided adhesive tape, and were then made electrically conductive by being coated with platinum (6 nm/min) using an EMI Tech Ion Sputter system (K 575 K) under vacuum (8 × 10^−3^ mbar) for 4 min at 15 mA. Microscopic images were then taken from different angles.

#### 2.5.2. Particle Size Analysis

The particle size distributions were measured using a laser diffraction particle size analyzer (Mastersizer 3000, Malvern Instruments, Malvern, UK). The particle size was expressed as Dx10, Dx50, and Dx90, taken from the cumulative distribution, where Dx10, Dx50, and Dx90 are the particle diameters calculated respectively at the 10th, 50th and 90th percentiles of undersized particles. The measurements were carried out in triplicate.

#### 2.5.3. Differential Scanning Calorimetry (DSC)

The thermal characteristics of the free MTX powder, carriers, PM, and SA-SD formulation were analysed using a DSC Q20 (TA Instruments; New Castle, DE, USA). In brief, 5 mg of each sample were sealed in the Tzero pan and lid, and heated from 60 °C to 175 °C at a constant rate of 10 °C/min with a nitrogen purge of 50 mL/min. The PM was prepared by mixing the drug and carriers in the same weight ratio as that of the optimized formulation.

#### 2.5.4. Powder X-ray Diffraction (PXRD)

The crystallinity of each sample was investigated at room temperature using a PXRD instrument (D/MAX-2500, Rigaku, Japan) with monochromatic Cu˗Kα radiation (λ = 1.54178 Å, 40 kV, and 100 mA) in the region of 5° ≤ 2θ ≤ 45°, and with an angular rise of 0.02°/s.

#### 2.5.5. Fourier Transform Infrared Spectroscopy (FTIR)

The FTIR patterns of the pure MTX, PM, and the formulation were obtained by FTIR spectrophotometer (FTIR-4100, JASCO, Pittsburgh, PA, USA). All of the samples were appropriately loaded onto the sample disk, and the resulting peaks were analysed using Spectra Manager II software in the 4000–400 cm^−1^ spectral range.

### 2.6. Pharmacokinetic Studies

#### 2.6.1. Care of the Experimental Animals

Twelve male Sprague–Dawley rats (Nara Biotech., Seoul, Korea), 7–8 weeks old and weighing 270 ± 20 g, were used for the in vivo pharmacokinetic studies. All of the animals were caged in controlled conditions of 23–25 °C/50–55% relative humidity (RH), and were freely provided with standard laboratory food and water. The protocols for the animal studies were also approved by the Institutional Animal Care and Use Committee at Hanyang University (permit number: 2014-0190A).

#### 2.6.2. Oral Administration and Blood Sampling

The rats were randomly divided into two groups of 6 rats per cage and left to acclimatize to the controlled conditions for 3 days. Prior to the pharmacokinetic experiment, the animals were fasted for 12h, but were allowed free access to water. Each rat was anaesthetized with diethyl ether and fixed on a surgical board. For cannulation, a polyethylene tube containing 50 IU/mL heparin in saline was placed into the right femoral artery of the rat. Then, each of the rats was orally administered with 1 mL aqueous suspension of MTX powder or MTX-loaded solid SASD at a dose of 20 mg/kg using an oral gavage. At predetermined time intervals, ~0.3 mL blood was collected via the cannulated tube and transferred into microtubes containing heparin, which were then centrifuged at 3000× *g* for 10 min to isolate the plasma.

#### 2.6.3. Sample Preparation and Statistical Data Analysis

To the collected plasma samples (90 μL), 100 μL methanol and 10 μL internal standard solution i.e., theophylline (1 mg/mL in methanol) was added. The mixture was vortexed for a few seconds and then centrifuged at 13,000× *g* for 10 min, and the supernatant was immediately transferred to vials for quantification by HPLC, as described in Section 2.2. A six-points calibration plot was constructed by the analysis of the working solution over the range 25–2000 ng/mL, and the response was linear throughout the range with R^2^ = 0.997. Moreover, the intra-day and inter-day variations were within the applicable limits (R^2^ = 0.998). The pharmacokinetic parameters of the area under the blood concentration–time curve (AUC), maximum plasma concentration (C_max_), time to reach the C_max_ (T_max_), half-life (t_1/2_), and elimination rate constant (K_el_) were derived using the WinNonlin software (Pharsight Corp., Mountain View, CA, USA). The values are reported as mean ± SD (*n* = 6), and the data were considered statistically significant at a *p*-value lower than 0.05 (*p* < 0.05) between the two formulations, as determined with Student’s *t*-test.

### 2.7. Photostability Study

The photostability test was determined as described by the ICH guidelines. Free MTX powder and MTX-loaded SASD formulation were spread over the bottom surface of chemically-inert, clear glass vials. These samples were then placed in a photostability chamber (Caron Model 6545-2, Caron, Marietta, GA, USA) maintained at 25 °C/60% RH and exposed to a light source of 1.2 million lux·hr intensity for 20 days. The drug content in the sample was determined at predetermined time intervals with HPLC, as described. The samples were also visually examined at the end of the study period for any physical changes.

## 3. Results and Discussion

### 3.1. Selection of the Carriers

The degree to which the drug and carriers are miscible strongly affects the solubility characteristics of the SD system [37]. Therefore, in order to derive the maximum solubility advantages from the solid dispersion system, impart a stronger drug–carrier interaction at molecular level, and prepare a stable SD system, the judicious selection of carriers is crucial. In order to determine the most suitable carriers, different hydrophilic polymers and surfactants were subjected to aqueous solubility screening.

The aqueous solubility of MTX in different polymers and surfactants is demonstrated in Table 2. Among the various hydrophilic polymers screened, Na-CMC with 710 µg/mL showed the maximum drug solubility, which was significantly higher than those of the other polymers. The solubility of MTX in water alone was approximately 65 µg/mL, which was expected given the hydrophobic nature of the drug. Specifically, the aqueous solubility of MTX in the presence of Na-CMC was increased approximately 11-fold over that in pure water. Similarly, of the surfactants screened, the solubility of MTX in SLS was significantly higher (4400 µg/mL) and in Cremophor EL was significantly lower. Based on these observations, Na-CMC and SLS were chosen as the carriers to prepare the ternary MTX-loaded SDs.

The significance of hydrophilic carriers—specifically polymers and surfactants for solubilization of hydrophobic drugs in a developing a ternary SD system—is thoroughly described in the literatures [38,39]. In brief, when SD comes in contact with the dissolution medium, a supersaturated state is rapidly achieved. However, the drug in this state is relatively unstable, and tends to de-supersaturate by precipitation into a large and more energetically-favourable form, which could decrease the dissolution and overall oral bioavailability [40,41]. The supersaturated state must therefore be maintained in order to achieve better solubility and maximum drug release. In these circumstances, the polymers of SD systems inhibit the drug precipitation by increasing the viscosity and restricting the drug’s molecular mobility within the drug–carrier matrix. In addition, the hydrophilic polymer adhered to the crystal lattice of the hydrophobic drug molecule in SASD systems acts as a mechanical barrier to restrict the aggregation of nuclei units, resulting in reduced crystal growth and enhanced solubility and drug dissolution [32].

### 3.2. Preparation and Optimization of MTX-Loaded SASD

Organic solvents are commonly used in SD, with both the drug and carriers being dissolved in an organic solvent, which is then evaporated using spray-drying, vacuum-drying, freeze-drying, spray-freeze-drying, rotary evaporation, or heat-drying in order to obtain the final solid mass. The use of organic solvents for the development of SD on an industrial scale is not ideal, given that they are hazardous or toxic to health and the environment, explosive in nature, and required in relatively large quantities to dissolve the drug, which increases the overall production cost [14]. In addition, the complete removal of some organic solvent/s is difficult to accomplish, and the presence of residual solvent clogged within the SDs can destabilize the SD system [38]. Here, we used an aqueous medium to prepare the MTX-loaded SASD. A fixed amount of the NA-CMC and SLS were first dissolved in water to form a solution, and a relatively large amount of hydrophobic MTX was dispersed, after which the suspension was spray-dried in order to fabricate the SASD. The final solid powder was free from toxic organic solvents, had a higher drug/carrier load, and did not require secondary drying steps.

**Figure 2:**
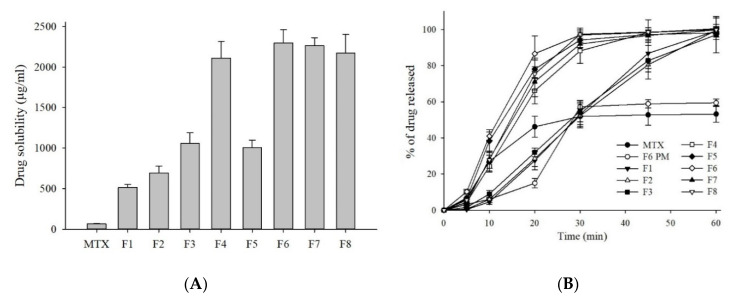
Drug solubility (**A**) and dissolution profiles (**B**) of MTX powder and MTX-loaded formulations. Each value represents the mean ± SD (*n* = 3).

In order to determine the optimum drug–carrier formula, we prepared different formulations by keeping the concentration of the drug constant and altering the amount of carrier (formulations F1–F4), or by changing the total weight ratio of the drug to the carrier (formulations F4–F8). These MTX-loaded SASD formulations were subjected to aqueous solubility testing; the results are shown in shown in Figure 2A. The aqueous solubility of MTX increased in the following order: F6 > F7 > F8 > F4 > F3 > F5 > F2 > F1. Of the eight formulations, the ternary SASD formulation—i.e., F6 prepared with MTX/Na-CMC/SLS at 3/0.5/0.5 (*w*/*w*/*w*)—resulted in the maximum drug solubility (2294 µg/mL). The binary SASD formulation—i.e., F1 composed of MTX and Na-CMC only at 3:1.5 (*w*/*w*)—improved the solubility the least (513 µg/mL). Interestingly, the solubility of the F6 formulation prepared at a relatively lower Na-CMC/SLS ratio (0.5/0.5) was actually higher than that of the ternary SD formulations prepared with higher drug/carrier ratios, i.e., F4 (0.75/0.75), F7 (1:1), and F8 (1.5/1.5). These findings indicate that higher carrier concentration does not always ensure greater drug solubility, and it is thus important to find an ideal drug–carrier concentration in order to maximize the solubility advantages of the SASD system.

### 3.3. In Vitro Dissolution Test

For drugs with poor water solubility, it is well known that improved drug solubility often results in an enhanced drug release and therefore increased oral bioavailability. The in vitro dissolution testing was performed in order to determine the dissolution profiles of the MTX, PM, and MTX-loaded SASD formulations; the results are shown in Figure 2B. As predicted, all of the SASD formulations showed improved drug release profiles over the free MTX powder. Specifically, formulation F6 showed almost complete drug release (ca. ~100%) within 60 min, with an overall drug dissolution approximately 1.9-fold higher than the free MTX. In contrast, the free MTX powder and F6 physical mixture (PM) showed low and incomplete drug dissolution in water, with the cumulative MTX release from the MTX powder and PM at 60 min being ca. 53% and 59%, respectively. Based on these results, a minor increase in the dissolution profile observed with PM indicates that the hydrophilic carrier (Na-CMC and/or SLS) itself could act as a solubilizer in these existing formulations, leading to a slight improvement in the drug dissolution.

More so, a burst drug release was observed in the F6 formulation, with a release of more than ca. 85% of the MTX within the first 20 min of the dissolution test. This increased and accelerated dissolution behaviour was mostly likely due to the adsorption of hydrophilic carriers onto the surface of the poorly water-soluble MTX molecules, resulting in prompt water uptake and improve wetting, and therefore accelerated drug release from the formulation [32,42]. In addition, the overall increase in the dissolution profiles observed with the spray-dried formulations can be ascribed to an increase in the effective surface area available for drug dissolution obtained by reducing the size of the particles and improving the saturation solubility [43].

As shown in Figure 2, the aqueous solubility and dissolution kinetics of MTX-loaded SASD were significantly improved by increasing the surfactant concentration in the Na-CMC/SLS ratio (Table 1). The addition of a third component, such as a surfactant, to create a ternary SD system facilitated drug solubilization by maintaining or lowering the degree at which supersaturation was achieved, preventing nucleation and thermodynamic crystal growth, thereby inhibiting drug precipitation and promoting drug leaching from the SDs, leading to improved solubility and dissolution [44,45]. Previous studies have reported that surfactants were effective as precipitation inhibitors and dissolution enhancers for several hydrophobic drugs, including celecoxib [44], atorvastatin [13], and tacrolimus [46]. Here, there was no substantial improvement in drug dissolution as the drug/carrier ratio was increased from 3:1 (F6) to 3:3 (F8). These findings reflected the carrier-mediated drug dissolution from the SASD. Relying on the solubility and in vitro dissolution kinetics, the formulation F6 with MTX/Na-CMC/SLS at a 3:0.5:0.5 (*w*/*w*/*w*) was chosen as an optimized formulation for further studies.

### 3.4. Morphological and Solid-State Analysis of the MTX-Loaded SDs

**Figure 3:**
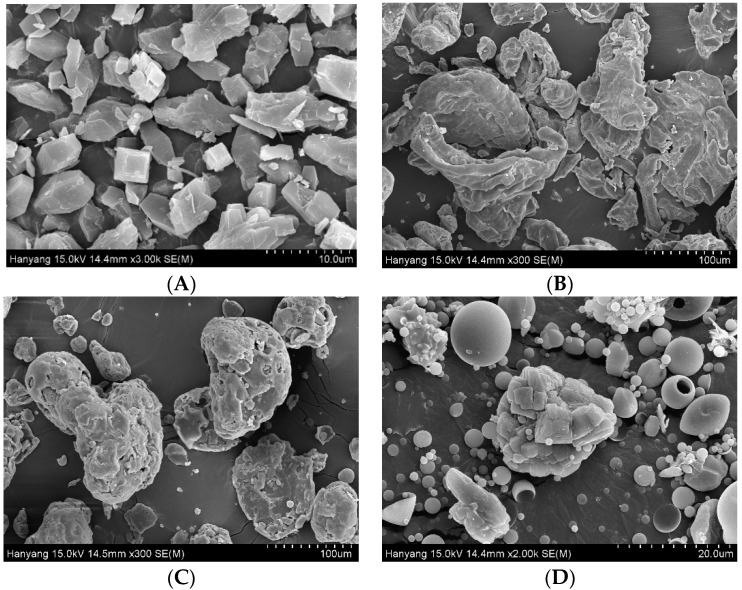
SEM images of (**A**) MTX powder (×3000), (**B**) Na-CMC (×300), (**C**) SLS (×300), and (**D**) the F6 formulation (×2000).

Scanning electron micrographs of the MTX powder, Na-CMC, SLS, and F6 formulation are shown in Figure 3. The surface topography of the MTX powder tends to have long, angular, crystal-shaped particles with sharp edges (Figure 3A). Moreover, the SEM images of Na-CMC (Figure 3B) and SLS (Figure 3C) show bulky, irregularly-shaped, rough-surfaced particles. Unlike the drug and excipients, the SASD formulation (F6) showed some relatively coarse-surfaced particles with non-round shapes, suggesting that the hydrophilic carriers might have adhered to the surface of the drug particles (Figure 3D). Noticeably, the SEM image of F6 also shows tiny, spherical, slightly smooth-surfaced particles typical of spray-dried formulations, indicating that the hydrophilic carriers might have coated or absorbed the hydrophobic drug particles during the spray-drying.

In the efficient production of SDs, particle size is crucial, as it greatly affects the in vitro/in vivo performance of the developed formulation. Hence, the particle size and distribution of the MTX powder and F6 formulation were analyzed. The median diameters, Dx50, were found to be 14.03 ± 2.97 µm and 7.15 ± 0.08 µm for the MTX and F6 formulation, respectively, which are significantly different from each other (Student’s *t*-test, *p* < 0.05). The Dx10 and Dx90 values of the tested samples of the MTX powder were 3.60 ± 0.48 µm and 103.73 ± 12.13 µm, respectively, and of the F6 formulation were 1.87 ± 0.04 µm and 29.55 ± 2.47 µm, respectively. Therefore, the results suggest that the improved solubility and dissolution of the F6 formulation is the outcome of its smaller particle size and narrow size distribution.

**Figure 4:**
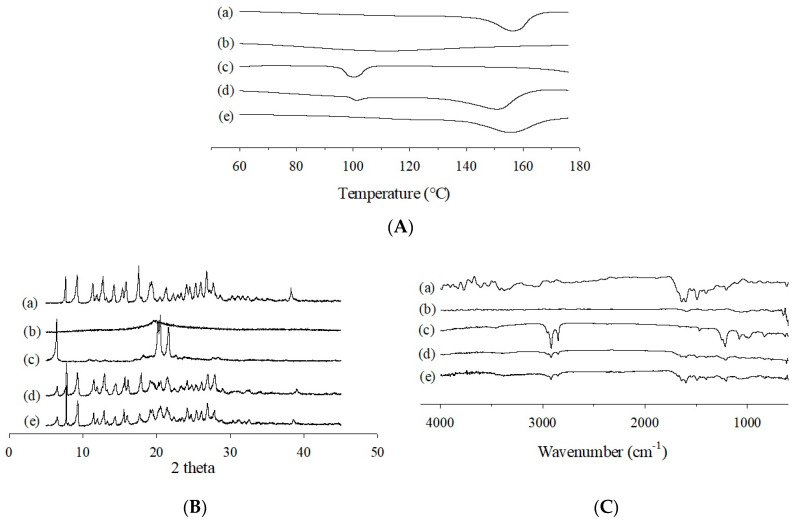
DSC (**A**), PXRD (**B**), and FTIR (**C**) curves of the MTX powder (**a**), Na-CMC (**b**), SLS (**c**), physical mixture (**d**), and F6 formulation (**e**).

The thermal characteristics of the MTX powder, Na˗CMC, SLS, PM, and F6 formulation were investigated using DSC (Figure 4A) and PXRD (Figure 4B). The DSC curve of free MTX powder revealed a slightly broad and distinct endothermic peak at about 150–160 °C, which makes sense given its melting point and crystalline nature (Figure 4(Aa)). Furthermore, the PM showed two small, broad endotherms at about 100 °C and 150 °C, which correspond to the endothermic peak of SLS and the melting point of the MTX, respectively (Figure 4(Ad)). Moreover, the characteristic endothermic peak of free MTX was also observed for the MTX-loaded SASD, which suggests that the drug was still in a crystalline structure and had not undergone complete amorphization (Figure 4(Ae)).

Additionally, the X˗ray diffraction curves of MTX revealed numerous sharp characteristic peaks, primarily due to its highly crystalline nature (Figure 4(Ba)). The representative peaks of the drug were also present in the X˗ray diffractometric profiles of the PM (Figure 4(Bd)) and the MTX-loaded SASD (Figure 4(Be)), which further verifies that the drug was in its crystalline state and had not undergone amorphous transition during the spray-drying with the aqueous solvent.

The physicochemical interactions between the drug and the carriers were investigated with FTIR spectroscopy. As shown in Figure 4(Ca), the major characteristic peaks of MTX were observed at about 3450 cm^−1^ (O–H stretching), 3050 cm^−1^ (N–H stretching), 1670–1600 cm^−1^ (C=O stretching from the carboxylic group and amidic group, resulting in a split band), 1500 cm^−1^ (N–H bending), and 1400 cm^−1^ (–C–O stretching). All of the bands observed in the FTIR spectrum were in strong alignment with the MTX molecular structure, and affirm its purity [36]. The FTIR spectra of PM included nearly every characteristic absorption band of the drug and carriers, with some peaks being superimposed, indicating the absence of interactions between the drug and carriers in the mixture (Figure 4(Cd)). Nevertheless, in the IR spectrum of MTX-loaded SASD, some of the distinguishing drug peaks were either completely masked or slightly shifted to a lower or higher wavelength, with reduced intensity (Figure 4(Ce)). These changes in the FTIR spectra suggest an interaction, such as inter-molecular hydrogen bonding, between the drug and SD carriers in the SASD formulation; however, a more detailed analyses should be performed to confirm this.

### 3.5. Pharmacokinetics Study

**Figure 5:**
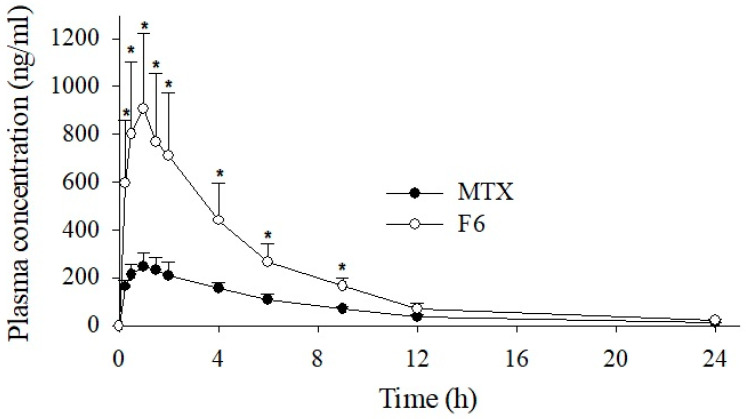
Plasma concentration–time profiles of MTX after the oral administration of the free drug or F6 formulation in rats. Each value represents the mean ± S.D. (*n* = 6). * *p* < 0.05 compared to free MTX.

The mean plasma concentration–time profiles of free MTX and the prepared MTX-loaded SASD are shown in Figure 5 and Figure S1 (log-scale), and the pharmacokinetic parameters after an oral administration of a dose equivalent to 20 mg/kg MTX are outlined in Table 3. As shown in Figure 5, the SASD formulation showed better drug absorption, resulting in a significantly higher plasma concentration (C_max_) and AUC compared to the free MTX powder, i.e., 906.27 ± 314.90 vs. 265.63 ± 57.05, and 4890.45 ± 1447.53 vs. 1738.71 ± 294.65, respectively. Specifically, the C_max_ and AUC of the SASD formulation were approximately 3.4- and 2.8-fold higher, respectively, than those of the MTX powder. Both the T_max_ and t half-life (t_1/2_) values of the formulation were shorter, and the elimination rate constant (K_el_) was slightly higher than for the free MTX powder. These in vivo pharmacokinetic data suggest that the prepared SASD formulation of MTX results in better drug absorption, leading to improved oral bioavailability. The in vivo pharmacokinetic findings align with those of the in vitro dissolution tests, and indicate that the improved bioavailability is the outcome of the enhanced solubility and higher drug release of MTX from the MTX-loaded SASD formulation. The overall oral bioavailability of the F6 formulation was increased 2.8-fold; the oral dose could be reduced 2.8-fold in order to achieve the same level of therapeutic response as that of the free MTX powder. In a clinical setting, this formulation strategy may provide several advantages, such as an enhanced therapeutic outcome at a reduced dose, the reduced use of excipients with reduced side effects, increased cost-effectiveness, and ultimately better patient compliance.

### 3.6. Stability Study

Light-sensitive active pharmaceutical ingredients (APIs) can be triggered by UVA/UVB or fluorescent light, and may undergo photochemical reactions, leading to photodegradation, the formation of toxic products [47,48], and loss of potency over time. It is therefore crucial to fabricate suitable formulations that can preserve and protect light-sensitive drugs during the production, storage, and processing of their dosage forms.

**Figure 6:**
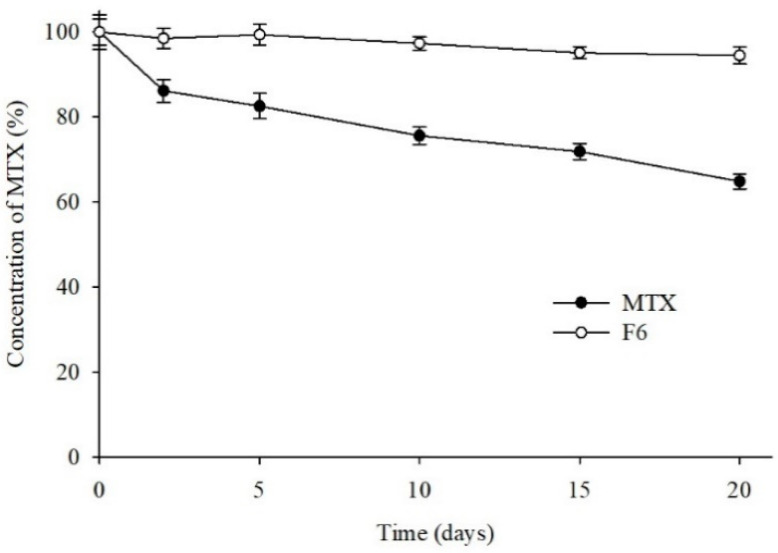
Photodegradation profile of MTX formulations. Each MTX sample was exposed to UV light (250 W/m^2^) for the indicated periods, and its purity was assessed by HPLC.

In this study, the photostability of MTX was assessed by a forced degradation test. The time-dependent changes in the MTX content when it was subjected to UV irradiation were examined for 20 days, and the MTX content was determined at set time intervals. Substantial deterioration only occurred with high exposure to artificial light. The results obtained after the forced photodegradation are shown in Figure 6. Even though ~65% of the MTX in the free MTX powder was found to be degraded by strong UV irradiation at the end of the study period, no significant/only slight photodegradation (5%) of MTX was detected in the MTX-loaded SASD. Furthermore, a distinct change in the physical appearance of the drug powder was observed, with the free MTX powder turning from a pale yellow to a light orange, indicating the photolytic degradation of MTX powder but not of formulation powder. In the F6 formulation, the carriers might have adhered to the surface of the photo-unstable drug (MTX) without altering its crystalline state, which might increase the drug’s photostability. SASD, therefore, might enhance the solubility, dissolution, and oral bioavailability of MTX without a crystalline change, leading to significantly-higher photostability. Moreover, compared to our previous study with MTX-loaded solid SMEDDS [36], we were able to achieve higher drug solubility and bioavailability with this SASD formulation (AUC 4890.45 ± 1447.53 vs. 3542.69 ± 670.73). Therefore, the SASD technique could be an effective approach for the improvement of the biopharmaceutical performance of poorly water-soluble drugs like MTX.

## 4. Conclusions

In this study, several spray-dried surface-attached SDs were successfully prepared with an aqueous medium to enhance the solubility, dissolution behavior, photostability, and oral bioavailability of a poorly water-soluble and photo-unstable drug, MTX. All of the in-house prepared SASDs showed significantly improved solubility and in vitro drug release kinetics over the free MTX powder. The in vivo pharmacokinetic studies of the optimized formulation in rats showed considerably higher plasma drug concentrations than the drug powder, mainly due to the enhanced drug dissolution; this resulted in improved GI absorption, and therefore higher bioavailability. Furthermore, it is clear from the photostability study that the MTX SASD system remains largely stable under UV irradiation, with insignificant physical changes. The DSC and PXRD affirmed that the drug remained intact in its fine crystalline state within the SASD formulation, while the FTIR indicated a possible interaction (hydrogen bonding) between the drug and the SD carriers. However, further studies are required in order to gain new insights into the molecular-level interactions between the drug and the SD carriers. Moreover, the safety profiles of the developed formulation are worthy to be explored in future work. Overall, these results suggest that the fabrication of SASD by spray-drying without an organic solvent could be a viable strategy to enhance the dissolution, bioavailability, and photostability of MTX and other lipophilic and unstable drugs.

## Figures and Tables

**Table 1:** Compositions of the surface-attached solid dispersions with different carrier ratios.

Composition	F1	F2	F3	F4	F5	F6	F7	F8
MTX (g)	3	3	3	3	3	3	3	3
Na-CMC (g)	1.5	1.25	1	0.75	0.3	0.5	1	1.5
SLS (g)	0	0.25	0.5	0.75	0.3	0.5	1	1.5

**Table 2:** Effect of carriers on the drug solubility. Each value represents the mean ± SD (*n* = 3).

Drug and Carriers	Drug Solubility (µg/mL)
Methotrexate (MTX)	65.40 ± 5.73
Hydrophilic polymers (1% aqueous solution)	
Polyvinylpyrrolidone (K 30)	51.22 ± 6.01
Sodium carboxymethyl cellulose	709.85 ± 22.81
Gelatin	406.54 ± 19.75
Polyvinyl alcohol	155.73 ± 6.97
Hydrooxypropyl cellulose	111.76 ± 11.13
Hydroxypropylmethyl cellulose	186.02 ± 54.03
Surfactants (10% aqueous solution)	
Tween 80	294.70 ± 24.61
Cremophor EL	55.39 ± 3.50
Cremophor RH40	158.10 ± 4.73
Transcutol P	133.07 ± 17.58
Sodium lauryl sulfate	4396.08 ± 171.90
Plurol diisostearique	936.34 ± 41.74
Capryol 90	62.82 ± 2.69

**Table 3:** Pharmacokinetic parameters of MTX powder, and MTX-loaded SASD formulation (F6).

Formulations	MTX	F6
AUC (h·ng/mL)	1738.71 ± 294.65	4890.45 ± 1447.53 *
C_max_ (ng/mL)	265.63 ± 57.05	906.27 ± 314.90 *
T_max_ (h)	1.13 ± 0.23	0.92 ± 0.20
t_1/2_ (h)	4.04 ± 0.50	3.28 ± 0.60
K_el_ (h^−1^)	0.17 ± 0.02	0.22 ± 0.04

* *p* < 0.05 compared with free MTX. Each value represents the mean ± S.D. (*n* = 6).

## Data Availability

The data presented in this study are available in the paper or in the supplementary material here.

## Supplementary Materials

The following are available online at https://www.mdpi.com/1999-4923/13/1/111/s1, Figure S1: Plasma concentration (log scale)–time profiles of MTX after the oral administration of the free drug or F6 formulation in rats.
