# Supplementary material for: Improved Bioavailability and High Photostability of Methotrexate by Spray-Dried Surface-Attached Solid Dispersion with an Aqueous Medium
Source: Pharmaceutics. 2021 Jan 16;13(1):111. doi: 10.3390/pharmaceutics13010111 (PMC7830624; doi:10.3390/pharmaceutics13010111)
Supplement: Supplementary file 1 [file pharmaceutics-13-00111-s001.pdf]

pharmaceutics

Supplementary data

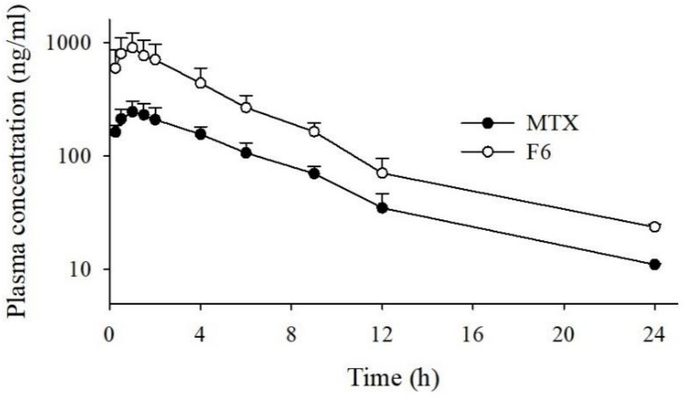

Figure S1. Plasma concentration (log scale)–time profiles of MTX after oral administration of free drug or F6 formulation in rats.
